# Delving Into the Origin of Destructive Inflammation in COVID-19: A Betrayal of Natural Host Defense Peptides?

**DOI:** 10.3389/fimmu.2020.610024

**Published:** 2021-01-22

**Authors:** Rebeca Garcia-Fandino, Ángel Piñeiro

**Affiliations:** ^1^Centro Singular de Investigación en Química Biolóxica e Materiais Moleculares (CIQUS), Departamento de Química Orgánica, Universidade de Santiago de Compostela, Santiago de Compostela, Spain; ^2^Departamento de Física Aplicada, Facultade de Física, Universidade de Santiago de Compostela, Santiago de Compostela, Spain

**Keywords:** COVID-19, SARS-CoV-2, antimicrobial peptides, lipid alteration, cell membrane, immune response

## Abstract

In contrast to other pathogenic agents that directly destroy host cells and tissues, the lethal power of SARS-CoV-2 resides in the over-reactive immune response triggered by this virus. Based on numerous evidences indicating that the lipid composition of host membranes is dramatically affected by COVID-19, and in the fact that our endogenous antimicrobial peptides (AMPs) are sensitive to the membrane composition of pathogenic agents, we propose that such destructive immune response is due to the direct action of AMPs. In a scenario where most host cell membranes are dressed by a pathogenic lipid composition, AMPs can indiscriminately attack them. This is why we use the “AMP betrayal” term to describe this mechanism. Previously proposed cytokine/bradykinin storm mechanisms are not incompatible with this new proposal. Interestingly, the harmful action of AMPs could be prevented by new therapies aimed to reestablish the lipid composition or to inhibit the action of specific peptides.

## Introduction

Severe acute respiratory syndrome–coronavirus 2 (SARS-CoV-2) is a single-stranded positive-sense RNA virus responsible for the coronavirus disease 2019 (COVID-19) global pandemic. Its name comes from its crown-like appearance under the electron microscope (1–3). Prior to SARS-CoV-2, six coronaviruses were known to cause diseases in humans, four of them provoking only mild to moderate symptoms and other two (SARS-CoV and MERS-CoV) leading to severe respiratory syndromes (4). Coronaviruses share a common spherical structure enclosed by a lipid bilayer. A large number of partially protruding proteins decorates the viral envelope. The typical diameter of coronaviruses ranges from 50 to 200 nm (5). Both SARS-CoV and SARS-CoV-2 use angiotensin-converting enzyme 2 (ACE2) as a cellular entry receptor, which is expressed mainly in the type II surfactant-secreting alveolar cells of the lungs, but also in most of human tissues, such as oral mucosa and gastrointestinal tract, kidney, heart, blood vessels, etc (6). Upon infection, SARS-CoV-2 typically penetrates external mucous membranes (nose, eyes and/or mouth) to subsequently access internal organs, mainly the lungs. Many individuals are apparently asymptomatic or they are recovered from mild symptoms within 1–2 weeks (7–10) but severe respiratory syndromes are often manifested. Patients with different chronic pathologies (diabetes, hypertension and cardiovascular disease) are especially susceptible to this virus (11). A large and still growing number of symptoms were ascribed to COVID-19, the most evident being shared with other coronavirus infections: fever (87.9%), cough (67.7%), fatigue (38.1%) and, to a lesser extent, diarrhea (3.7%) and vomiting (5.0%) (3, 12). Additional common symptoms ascribed to SARS-CoV-2, which are typically reversible within 2–4 weeks after infection, are the loss of smell and taste (13). However, in contrast with other coronaviruses, COVID-19 frequently exhibits unexpected long-term severe consequences (5, 14). Patients recovered from the severe form of the disease, and even those who had mild and moderate symptoms present abnormal findings on cardiovascular MRI (on average up to 71 days after diagnosis), dyspnea, unusual fatigue, muscle weakness headaches, memory lapses, changes in mood, sleep difficulty, palpitations, needle pains in arms and legs, etc.

## The Innate Immune Response in Covid-19: Cytokine and/or Bradykinin Storms?

Immune response against strange pathogenic agents is not a trivial process. A battery of defensive resources is available to protect organisms from potentially toxic invasions, which can be classified in those belonging to the innate and to the adaptive immune systems. These two defensive barriers are not independent but they are closely connected to each other (15). Both consist of complex networks of cells, signaling molecules and regulatory pathways. The innate immune response is involved in the activation of adaptive immunity, and has a critical role in controlling infections during the period of 4–7 days before the initial adaptive immune response takes effect (16). It has been reported that the adaptive immune system is key to explain the large amount of asymptomatic infected people. Mateus et al. (17) suggested that T cell response to SARS-CoV-2 without previous exposition to the virus could be due to the presence of homologous epitopes from a common cold coronavirus. On the other hand, hospitalized patients exhibit different T cell response patterns, which could be related with different degrees of severity of the disease and also would be relevant to decide an appropriate treatment (18). The adaptive immune response is also important for vaccine development as well as to establish pandemic control measurements (19).

Most infectious agents induce inflammation by activating innate immunity. Time response and coordination of the different immune defensive barriers are key to protect against pathogenic agents. Immune overreaction may lead to uncontrolled swelling of the affected tissues. Interestingly, a similar dysfunctional immune reaction, accompanied by tissue inflammation, has also been reported for several systemic diseases and cancer (20). To date, our understanding of the specific innate immune response to SARS-CoV-2 is not complete. It is widely accepted that the high mortality in COVID-19 is not directly caused by the virus but by the abovementioned innate immune response that provokes destructive inflammation (21, 22). Numerous studies reveal that the level of several inflammatory cytokines is abnormally high in serum as well as in different organs, suggesting that they play a significant role in COVID-19 pathogenesis (23, 24). Several life-threatening respiratory symptoms of COVID-19 such as plasma leakage, vascular permeability, and disseminated vascular coagulation have been attributed to this hypercytokinemia, also called “cytokine storm”. Cytokine storms have been associated with a wide variety of infectious (SARS-CoV-1, MERS, avian flu, etc) and noninfectious diseases. They have even been observed as a result of clinical trials with monoclonal antibodies able to stimulate T cells (25, 26). This out-of-control immune response exhibits a double-sided character, on the one hand it magnifies the danger signal of the virus invasion and on the other hand it provokes a destructive inflammation as well as the host cell damage. The molecules released from the destroyed cells, specially DNA from stressed mitochondria, cardiolipin, cytochrome C and segments of nuclear DNA, are recognized as damage associated molecular patterns (DAMPs) by molecules from the intra and intercellular immune system (mainly TLR4 TLR7, TLR9, and cGAS). In turn, this induces the massive release of proinflammatory cytokines leading to a secondary cytokine storm. This feedback process becomes cyclic and it eventually results in irreversible damage of tissues by apoptosis, pyroptosis and necrosis even of non-infected cells. The suicide of non-infected cells could be a defensive strategy to obstruct the propagation of the virus but the final balance of all these competitive processes (virus propagation and cell death) could become negative (27). Thus, there is a subtle harmony between protective and pathogenic immune reaction upon coronavirus infection. Controlling the local and systemic inflammatory response in COVID-19 and dampening the devastating overreaction of the immune system may be as important as antiviral therapies. Immunomodulatory drugs have been proposed as a treatment to address the immunopathology of COVID-19 infection. A clear advantage of this approach is that it is not specific against a given virus strain, i.e. it is not sensitive to mutations. In practice, this strategy is difficult to implement due to the limited understanding of the multidimensional coupled compounds of the immune system. To date, attempts to develop treatments in this direction were not successful (28). Moreover, this kind of therapies are considered to be highly risky since anti-cytokines could interfere with antiviral natural responses or pharmacological treatments (22).

A new mechanism called “Bradykinin storm” has been recently proposed as a non-exclusive alternative to cytokine storms (29, 30). Based on the analysis of samples collected from the lungs of patients with COVID-19, this mechanism considers the antagonist action of ACE and ACE2 to regulate the blood pressure. The authors of this proposal claim that the renin-angiotensin system (RAS) and the kinin-kallikrein pathways are altered by the virus, resulting in a decreased expression of ACE (a natural bradykinin breaker) together with an increased expression of ACE2 and the two bradykinin receptors, among other proteins. All this cooperates to the overexpression of bradykinin. It is known that this vasodilator peptide induces pain as well as an important alteration of the blood vessels: they are expanded and become leaky, causing swelling and inflammation of the surrounding tissue (29). The presence of hyaluronic acid, which is known to absorb relatively large amounts of water molecule forming a hydrogel, has also been found to be overexpressed. The leakage of fluid induced by the action of bradykinin combined to the presence of hyaluronic acid results in the gelling of the vessels, preventing the O_2_-CO_2_ exchange, and thus causing severe acute respiratory syndrome typically present in COVID-19 patients.

## Host Defense Peptides in Natural Host Immunity

A range of cells including neutrophil and macrophage phagocytic cells, epithelial cells, mast cells, eosinophils, and natural killer cells form part of the innate immune system. These structures are immediately available, so they are expected to respond rapidly to the presence of pathogen agents upon triggering their pattern recognition receptors (TLRs, C lectin and scavenger receptors). As a result, several elements are released and/or activated, namely cytokines, chemokines, superoxides, nitric oxides, prostaglandins, acute phase proteins, and antimicrobial peptides (AMPs) (31). AMPs, also known as host defense peptides, represent an essential part of the human immune system of virtually all organisms due to their broad spectrum activity against a wide range of pathogens, like bacteria, fungi, and viruses (32–34). Owing to their versatility, microbicidal capability, favorable pharmacokinetic properties, and low propensity for resistance development, AMPs are especially promising to deal with a number of infections, including COVID-19 and also emerging infections caused by viral pathogens for which no approved vaccines or treatments are currently available, such as dengue virus (DENV) and Zika virus (ZIKV) (35, 36). Thus, AMPs can be considered as endogenous antibiotics (37). They are produced and stored by epithelial and professional host defense cells such as macrophages, neutrophils or mast cells, among others. AMPs are abundant in a wide variety of highly vulnerable to pathogen tissues, including skin, eyes, oral cavity, ears, airway, lung, female reproductive tract, cervical-vaginal fluid, intestines, and urinary tract (38). Some cells such as neutrophils contain a high number of constitutive AMPs, but their expression can also be triggered by the presence of microbial or host stimuli. Consequently, the AMP profile (peptidiome) is highly variable depending on the location and host condition (20). Notably, the susceptibility to a virus infection depends on the type and amount of particular AMPs expressed by the individual (39). It has been observed that gut microbiome perturbation by different internal and external mechanisms may trigger an inflammatory overreaction in healthy individuals (40, 41). Interestingly, gut bacteria represent a major source of AMPs production in the gastrointestinal tract. A number of peptides and proteins able to stop the invasion of pathogenic microorganisms, including defensins, cathelicidins, C-type lectins, ribonucleases, and S100 proteins in intestinal epithelial cells and Paneth cells, are regulated by gut microbiome (42). Thus, there is a synergy or feedback process involving AMPs and microbiota, since the concentration and composition of the latter is regulated by AMPs. Mast cells, typically located in the submucosa of the respiratory tract and in the nasal cavity, have also been reported to be key in the host-microbiota information exchange, by triggering the release of AMPs (43). In fact, mast cells not only express different innate immune receptors, such as TLRs, that initiate pathogen recognition, but they can also be activated to directly kill pathogens by phagocytosis or through AMPs release (44–46). Interestingly, the important role of mast cells as a key part of the primary defense barrier in coronavirus infection has been recently observed, presenting a dual role in the disease (47). On the one hand, they contribute with other elements of the immune system to prevent the proliferation of the infection but they also favor the inflammation by releasing pro-inflammatory cytokines such as IL-1, IL-33, IL-18, and TNF.

At present, more than 2,000 peptides derived from animals have been identified, including ~130 of human origin: defensins, cathelicidins, transferrins, hepcidin, human antimicrobial proteins, dermcidin, histones, AMPs derived from known proteins, chemokines, and AMPs from immune cells, antimicrobial neuropeptides, and beta-amyloid peptides (48, 49). Most of them are small, cationic, amphipathic peptides with <50 amino acids and exhibit a diversity of structures and functions. Many of these peptides act directly on lipid cell membranes, without the need for specific membrane receptors (50–53), thus hindering the development of resistance mechanisms. Although a precise understanding of the relationship between AMP structure and their cytolytic function in a range of organisms is still lacking, there are numerous models to explain their action mechanism, including the so called toroidal, barrel-stave, and carpet models (54, 55). The interaction between AMPs and the target membrane is critical to the specificity and activity of these peptides. There is an important difference between the surface electrostatic charge of prokaryotic and eukaryotic cells due to the large abundance of anionic phospholipids in the former, compared to the dominance of zwitterionic and uncharged lipids in the latter (56). Notably, also the outer leaflet of cancer cells is negatively charged (57–61). Due to their cationic character, antimicrobial peptides have a preference for anionic membranes, typically presented by pathogens such as bacteria, enveloped virus, and even cancer cells. Thus, the interaction is hypothesized to be driven mostly by electrostatic interactions, although hydrophobic interactions are also expected to be important as a last resort. Membrane composition can therefore be exploited to design new antimicrobial and tumor cell destroying lytic peptides, since it is key for their activation and their action mechanism. This is, AMPs are membrane-composition specific and pathogenic agents share a range of lipidomic features that makes them suitable targets for these peptides.

## The Role of Lipids in the Infection by SARS-CoV-2: All Upside Down

Almost every day global scientific efforts reveal something new about SARS-CoV-2. Most of the effort has gone into sequencing the genome of the virus and studying the proteins that are present in its membrane. This is of unquestionable value. However, there is still scarce knowledge about the crucial paper that the lipids play in the infection (62). Since lipids are a key structural component of the most exposed region of cells and this virus, they are expected to play a central role during the infection. It has been proposed that SARS-CoV-2 can rapidly switch its membrane lipid structure-function (63). A quick exchange in the membrane composition at the exocytosis stage could explain the significantly different fatty acid profile in infected cells compared to that of the virus particles entering the environment. This adaptive skill of the virus protects it against strong changes of environmental conditions, thus maximizing its replication. It has been reported that coronaviruses take the control of endoplasmic reticulum-Golgi intermediate compartment and infected cells release mature virus entities as vesicles budding from the trans-Golgi network (64, 65).

On the other hand, changes in lipid host membrane composition triggered by different diseases, including viral infections, have been reported. It is known that some viruses cause significant change in the lipid composition of the host cell membranes. They also take the control of the cell metabolism, hijacking the host lipidome, to favor the propagation of the infection (66). This mechanism has also been identified in SARS-CoV-2 (62). Recent studies observed dyslipidemia in patients infected by this virus, indicating that blood lipid might be involved in the pathogenesis of COVID-19, and even suggesting that blood lipids may be considered as a potential and available indicator of COVID-19 severity (67). Ayres et al. (68) showed that the phospholipid profile of the bronchoalveolar lavage fluid in patients with acute respiratory distress syndrome had shown significantly low levels of phosphatidylglycerol. A lower level of palmitate acid had also been observed in the same individuals. These two molecules are exchanged by an increased concentration of minor components. It has been also observed that the development of hypolipidemia begins in patients with mild symptoms and it progressively becomes worse in an association with the disease severity (69). Shen et al. (70) found that over 100 lipids were downregulated in severe patients. Their data showed decreased sphingolipids in both non severe and severe COVID-19 patients. They found continuous decrease of glycerophospholipids after SARS-CoV-2 infection. Choline and its derivatives were downregulated, particularly in severe cases, while phosphocholine, the intermediate product for producing phosphatidylcholine (PC) was upregulated. It has been recently published that the serum lipid pattern of infected cells exhibits higher levels of sphingomyelins (SMs) and plasma monosialodihexosyl gangliosides GM3s (cell-type specific), and lower amounts of reduced diacylglycerols (DAGs), compared to cells of healthy patients. Such perturbation in lipid composition is similar to that of exosomal membranes (71). In the same line, a significant alteration of the membrane composition in red blood cells from patients with COVID-19, and in particular a reduction in the presence of short and medium chain saturated fatty acids, acyl-carnitines, and sphingolipids, was recently observed by T. Thomas et al. (72). For longer saturated fatty acids and acyls groups, palmitate (C16) and specially stearate (C18) the trend was in the opposite direction while the concentration of the long unsaturated C18:3 acyl also decreased. No significant changes were observed in other mono or poly unsaturated fatty acids. The most significant lipid alterations were observed for sphingolipids, CmE, lysophosphatidic acids, cPA and ceramide-phosphorylethanolamine, with a clear reduction of the former three and a subtle increase in the latter two. A clear concentration increase was observed for several lipids in infected red blood cells: mainly PE(30:3), PE(36:2), Hex2Cer(m31:1) and PC(34:2) (72). The lipid composition of blood plasma cells was also analyzed by D. Wu et al. (73). These authors found a correlation between the levels of metabolite and lipid alterations and the severity of the disease in the fatal cases. In particular, the concentration of diglycerides, free fatty acids and triglycerides increase with the advance of the infection, while that of phosphatidylcholines decreases. The lipid profile was observed to be significantly less altered in patients with mild to severe symptoms who finally recovered from the disease. However, even for these patients, their lipid profile did not return to normal even after the virus is undetectable and not apparent symptoms are present. This indicates that the full metabolic recovery after being hijacked by the virus (74), is much slower than that from other most evident symptoms (73). This idea is supported by other studies. Noteworthy, Ayres et al. (68) found several metabolic alterations, including hyperlipidemia, 12 years after infection by SARS-CoV. Interestingly, it has been noted that children affected by SARS-V-2 may develop a disease similar to Kawasaki’s illness, which, as happens in COVID-19, is mediated by pro-inflammatory cytokines produced by innate immunity cells (75). Unfortunately, no lipidemia studies have been reported in children with this pathology but it is known that Kawasaki disease produces persistent altered lipid metabolism (76–78). Overall, this suggests that membranes could be used as a target to develop new drugs although, to our knowledge, no treatments based on this strategy have been developed. It is interesting to mention that the human coronavirus 229E (HcoV-229E) has been used to characterize the change in lipid composition of infected cells. This analysis revealed that the concentration of glycerophospholipids and fatty acids was significantly increased. In particular, those of arachidonic and linoleic acids were remarkable. Interestingly, the addition of these two fatty acids to infected cells lead to a reduction in the replication of the virus. This inhibitory effect was also observed for MERS-CoV (13). Curiously, the metabolic and immune response of bats to the infection of viruses is different to that of other known mammals. They tolerate better the infection by avoiding the immune overreaction. Bat lipidomics has been studied in connection with several diseases. For instance, the lipid composition of bat wing epidermis has been analyzed to study its correlation with cutaneous infections by *Pseudogymnoascus destructans* during hibernation. A reduction in the amount of myristic and linoleic acids was observed in this period. These two acids, together with oleic and palmitoleic, inhibit the growth of this fungus. Thus, bats are more vulnerable to infection during hibernation (79).

## Betrayal of Host Defense Antimicrobial Peptides Amid Chaos: A Conflict in the Cell Membrane

**Figure 1:**
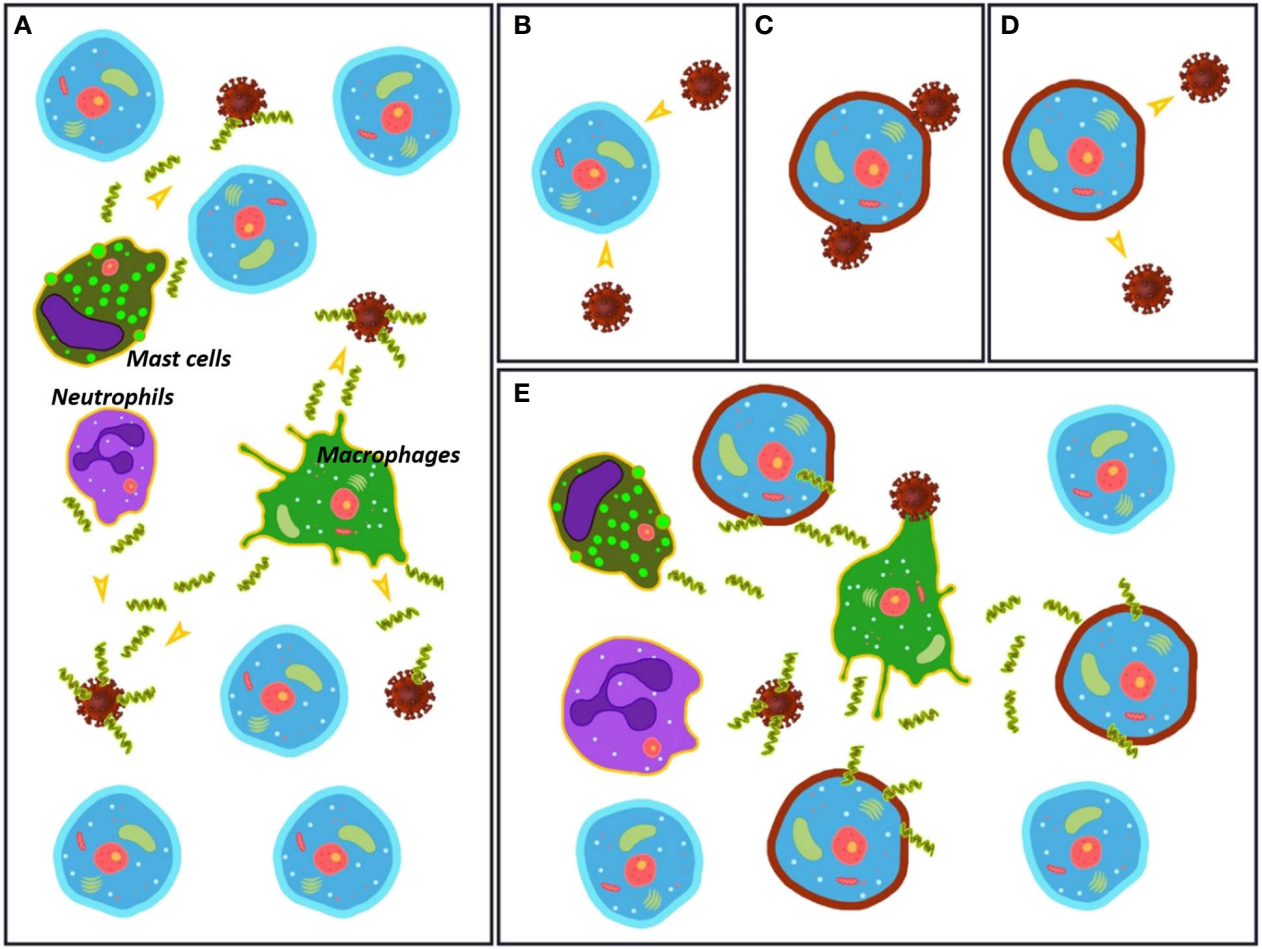
**(A)** Initial stage of the infection process with the virus in an environment rich in healthy cells. Macrophages, neutrophils and mast cells (among others) release antimicrobial peptides (AMPs) that act directly on the virus particles. **(B–D)** The virus changes the membrane composition of the host cells. The mechanism for this alteration is unknown. **(E)** The AMPs released by macrophages, neutrophils and mast cells (among others) attack both virus particles and damaged cells, triggering an immune overreaction.

So far, we have disclosed several facts related to SARS-CoV-2 infection and the uncontrolled overreactive innate immune response resulting in destructive inflammation. To these pieces of the puzzle, we must add that the alteration of various lipid species during COVID-19 infection is dramatic, together with the fact that the specificity exhibited by AMPs, key molecules in the innate immune response, relies on the different lipid composition between pathogen and host cells. Given the clear evidences that SARS-CoV-2 strongly perturbs the membrane composition of host cells, natural AMPs produced and stored by specialized defensive cells such as macrophages and neutrophils are expected to react. Their native mission is to recognize unspecific strange lipid patterns caused by infections, in order to destroy the corresponding cells. We hypothesize that the dramatic lipid alteration of the host cells caused by the virus could trigger the response of natural AMPs, activating the first line of defense toward host cells that should not be destroyed. This assumption brings a new player, natural AMPs, into the overreactive innate immune response observed in COVID-19, resulting in destructive inflammation. The change in membrane composition would cheat the natural defenders that act directly on the lipid cell membrane to indiscriminately attack the cells with an altered lipid composition. This action mechanism does not require the presence of protein receptors but it is expected to happen in a situation where the invading agent perturbs the environment by dressing all the cells with the same pathogenic coating. This proposal is illustrated in **Figure 1**. To our knowledge, although the great influence that the composition of the lipid membranes has on the mode of action of natural AMPs is known, the lipid alteration that takes place in the host cells during COVID-19 has not been related to the action of these natural host defense peptides.

The scope of this assumption goes beyond the infection by SARS-CoV-2 and makes sense also for other diseases. It is known that AMPs play a pathological role in several inflammatory diseases, cancer and even psychiatric disorders for which they have even been proposed as potential biomarkers. They have been identified as key elements in a number of autoimmune disorders, acting as potent modulators for both pro- and anti- inflammatory responses (80, 81). Our proposal might explain the connection between the lipid alteration triggered by infections and undesirable inflammation events resulting from the massive expression of AMPs. In this scenario, new therapies aimed to reestablish lipid composition or to block specific AMPs involved in host suicide missions could arise. Such alternative therapies are expected to be less aggressive and safer than the chemical inhibition of immune defensive response. To our knowledge no drugs have been specifically developed to target AMPs but some molecules have shown to affect their expression or/and activity, opening different avenues to design antiAMP drugs. For instance, dexamethasone is a glucocorticosteroid that proved to inhibit the expression of human cathelicidin, human beta defensin 1, lysozyme and secretory leukocyte peptidase 1 in the THP-1 monocytic cell-line (THP-1 monocytes) (82). Interestingly, it has been claimed that this drug may reduce mortality of severe COVID-19 patients. This effect has been associated to the decreased production of cytokines as well as to the inhibition of the protective function of T cells and to the block of B cells from making antibodies (83). Anti-AMPs have also been designed to optimize complementary coiled-coil interactions with AMPs (84). Using this strategy, the resulting superstructures become functionally inert. On the other hand, it has been shown that iron oxide nanoparticles inhibit AMP function (85), what can be exploited for therapeutic purposes. β-arrestin1 was also shown to down regulate AMP expression in shrimp, by interacting with TC45, tyrosine phosphatase of T cells (86). Finally, it has also been reported that several cytokines are able to inhibit the expression of AMPs (87). Some of these molecules could be taken as a reference to develop new drugs.

## Summary and Outlook

In this work, we have exposed some of the main evidences revealing the over-reactive innate immune response and the subsequent destructive inflammation caused by SARS-CoV-2 infection and also by other diseases. We have also compiled data on the dramatic lipid alteration that the virus causes in the membranes of host cells and we have related this alteration to the role of natural host defense peptides (AMPs), often ignored in the currently accepted cytokine or bradykinin storm mechanisms. As far as we know, the lipid composition alteration that takes place in the host membrane cells during COVID-19 had not been directly related yet to the action of AMPs, although it is well known that the action mechanisms of most of these peptides converge to the destruction of pathogenic membranes. We propose that the massive modification of the altered host membranes by the virus, widely documented, triggers the response of natural AMPs by destroying them as they do with the membranes of other pathogenic agents. This proposal could contribute to explain the first cause of death by COVID-19: acute respiratory failure due to the self-immune disruption of the lung cells. The reestablishment of lipid composition or the blockage of specific AMPs involved in the destruction of host cells could be considered as possible therapeutic intervention points.

## Data Availability Statement

The original contributions presented in the study are included in the article; further inquiries can be directed to the corresponding authors.

## Author Contributions

The manuscript was written through contributions of all authors. All authors contributed to the article and approved the submitted version.

## Funding

This work was supported by the Spanish Agencia Estatal de Investigación (AEI) and the ERDF (RTI2018-098795-A-I00) by the Ministerio de Ciencia e Innovación (PID2019-111327GB-I00), Xunta de Galicia (ED431F 2020/05 and Centro singular de investigación de Galicia accreditation 2019-2022, ED431G 2019/03) and the European Union (European Regional Development Fund - ERDF), and the National Portuguese Funds through FCT- Fundação para a Ciência e Tecnologia, and European funds through FEDER (project with reference PTDC/BIA-BFS/30579/2017; POCI-01-0145-FEDER-030579). RG-F thanks Ministerio de Ciencia, Innovación y Universidades for a “Ramón y Cajal” contract (RYC-2016-20335).

## Conflict of Interest

The authors declare that the research was conducted in the absence of any commercial or financial relationships that could be construed as a potential conflict of interest.
